# Clinical expert consensus document on rotational atherectomy from the Japanese association of cardiovascular intervention and therapeutics

**DOI:** 10.1007/s12928-020-00715-w

**Published:** 2020-10-20

**Authors:** Kenichi Sakakura, Yoshiaki Ito, Yoshisato Shibata, Atsunori Okamura, Yoshifumi Kashima, Shigeru Nakamura, Yuji Hamazaki, Junya Ako, Hiroyoshi Yokoi, Yoshio Kobayashi, Yuji Ikari

**Affiliations:** 1 Division of Cardiovascular Medicine, Saitama Medical Center, Jichi Medical University, 1-847 Amanuma, Omiya, Saitama City, 330-8503 Japan; 2 Department of Cardiology, Saiseikai Yokohama City Eastern Hospital, Yokohama, Japan; 3 Department of Cardiology, Miyazaki Medical Association Hospital, Miyazaki, Japan; 4 Division of Cardiology, Sakurabashi Watanabe Hospital, Osaka, Japan; 5 Division of Interventional Cardiology, Cardiovascular Medicine, Sapporo Cardio Vascular Clinic, Sapporo Heart Center, Sapporo, Japan; 6 Cardiovascular center, Kyoto Katsura Hospital, Kyoto, Japan; 7 Division of Cardiology, Ootakanomori Hospital, Kashiwa, Japan; 8 Department of Cardiovascular Medicine, Kitasato University School of Medicine, Sagamihara, Japan; 9 Department of Cardiology, Fukuoka Sanno Hospital, Fukuoka, Japan; 10 Department of Cardiovascular Medicine, Chiba University Graduate School of Medicine, Chiba, Japan; 11 Department of Cardiology, Tokai University School of Medicine, Isehara, Japan

**Keywords:** Rotational atherectomy, Calcification, Percutaneous coronary intervention, Intravascular ultrasound, Optical coherence tomography.

## Abstract

Rotational atherectomy (RA) has been widely used for percutaneous coronary intervention (PCI) to severely calcified lesions. As compared to other countries, RA in Japan has uniquely developed with the aid of greater usage of intravascular imaging devices such as intravascular ultrasound (IVUS) or optical coherence tomography (OCT). IVUS has been used to understand the guidewire bias and to decide appropriate burr sizes during RA, whereas OCT can also provide the thickness of calcification. Owing to such abundant experiences, Japanese RA operators modified RA techniques and reported unique evidences regarding RA. The Task Force on Rotational Atherectomy of the J apanese Association of Cardiovascular Intervention and Therapeutics (CVIT) has now proposed the expert consensus document to summarize the contemporary techniques and evidences regarding RA.

## Introduction

Severe calcification in atherosclerotic plaques has been the most common cause of poor clinical outcomes since the beginning of percutaneous coronary intervention (PCI) [1, 2]. Rotational atherectomy (RA) has been widely used for severely calcified coronary lesions for more than 20 years to improve clinical outcomes in patients with severely calcified lesions. Recently, North American expert review as well as European expert consensus on RA have been published to provide a clinical standard for RA operators [3, 4]. As compared to North America and European countries, RA in Japan has uniquely developed with the aid of greater usage of intravascular imaging devices [5, 6]. Since the cost of intravascular ultrasound (IVUS) or optical coherence tomography (OCT) during percutaneous coronary intervention (PCI) has been covered by the government insurance system in Japan, RA operators could easily access to IVUS or OCT. IVUS has been used to understand the guidewire bias and to decide appropriate burr sizes during RA [7], whereas OCT can be used to measure the thickness of calcification during RA [8], which could result in appropriate burr size up [9]. Furthermore, the prevalence of PCI with RA has been higher in Japan than in other countries. The prevalence of PCI with RA was approximately 3.3% in Japan [10], which was similar to those in United Kingdom (3.1%) or France (2.9%) and was higher than those in Italy (1.3%) or Germany (0.8%) [4].

On the other hand, RA has not been allowed to any operators without on-site surgical back-up in Japan since the beginning of RA. However, the government and the Japanese Association of Cardiovascular Intervention and Therapeutics (CVIT) worked together, and released the new facility criteria for RA in April 2020, which allowed operators to perform RA without on-site surgical back-up. The new facility criteria consisted of four components: [1] the facility must register all cases on J-PCI registry, [2] the new facility undergo a device training organized by CVIT and manufacturer, [3] if the facility had many complications or did not have sufficient cases, the facility would undergo a device training again under the CVIT recommendation, [4] RA should be performed or supervised by senior PCI operators who experienced more than 300 PCI cases, and severe complications regarding RA should be reported to CVIT. The new facility criteria would rapidly increase the number of RA operators in Japan. Although CVIT and manufacturer (Boston Scientific Japan) have organized a training program for new RA operators, this unique circumstance in Japan has increased a need for dedicated references, especially focused on junior RA operators. The purpose of this document was to summarize the contemporary techniques and evidences regarding RA and to provide an expert consensus document for RA operators.

## Aim of RA

Before the stent era, the main purpose of RA was to debulk atherosclerotic plaques including calcification in coronary arteries [11]. However, the incidence of restenosis was considerable following debulking using RA [12]. Emergence of drug-eluting stent (DES) has dramatically changed the indications of PCI, which included diffuse long calcified lesions. The lesion modification, which facilitate the delivery and expansion of DES, would be the most frequent purpose in the contemporary RA, and the long-term outcomes of DES following RA was acceptable [13–17], except specific lesions such as calcified nodule [18]. Moreover, RA might prevent the polymer damage, when DES was delivered to the calcified lesions [19]. Although the initial results of debulking using RA was not satisfactory, the debulking might have developed with the aid of imaging devices and drug-coated balloon in Japan [20–22]. The contemporary indications for RA are summarized in Table 1.

**Table 1:** Contemporary indications for rotational atherectomy

Definite indications	Severely calcified lesions (typically 360 degree calcification)
	Napkin ring calcification
	Calcification showing reverberation in IVUS
	Device uncrossable lesions
	IVUS/OCT could not cross (relatively common)
	Microcatheter could not cross (relatively rare)
Possible indications	Moderately calcified lesions (> 180 degree calcification)
High risk lesions	Lesions with thrombus
	Lesions with extensive dissection
	Lesions with an angle
	Bypass graft lesions
Contraindication	Last remaining vessel with compromised left ventricular function

*IVUS* intravascular ultrasound, *OCT* optical coherent tomography

## Junior RA operator

In this document, we defined junior RA operator as RA operator with less than 50 RA experiences. Because the number of RA cases per year in the facility was inversely associated with adverse events [10], the number of RA cases per operator would be important to prevent severe complications. Moreover, the good indication for RA is not necessarily low-risk. For example, the ostial right coronary artery (RCA) lesions have been recognized a good indication for RA [23], whereas RA to the ostial RCA is known to be technically difficult [24]. Therefore, the recommendation for junior RA operators may be different from that for senior operators in some sections.

## Patient’s general conditions, mechanical support, temporary pacing

When we plan to perform RA, we should evaluate patient’s general conditions such as vital signs and cardiac functions. Although there was no evidence regarding blood pressure during RA, it would be important to keep systolic blood pressure (SBP) ≥ 120 mmHg (at least ≥ 100 mmHg) for the prevention of complications such as slow flow. If slow flow occurred in patients with left ventricular dysfunction, there would be a greater risk of hemodynamic collapse. If a patient with left ventricular dysfunction shows low SBP, we may consider using mechanical supports such as intra-aortic balloon pumping (IABP) before RA. Although it is very rare to insert veno-arterial extracorporeal membrane oxygenation (V-A ECMO) for elective PCI with RA, it is an option for very high-risk PCI with RA to take additional arterial and venous sheathes just in case of emergent V-A ECMO. Moreover, if such patient would undergo elective RA, it is better to evaluate the abdominal and thoracic aorta by computed tomography (CT) to check the contraindications for IABP such as aneurysm or shaggy aorta. Although the Impella (Abiomed, Danvers, MA, USA) is not allowed to use as a support device for patients who undergo elective high-risk PCI in Japan, the Impella is considered to be an option as a support device for elective PCI with RA in USA [25]. If patients with cardiogenic shock already received the Impella or V-A ECMO supports, RA to the severely calcified lesions can be a bailout option from cardiogenic shock. Furthermore, although the beta-blockers are cornerstone for optimal medical therapy, some operators hesitate to use beta-blockers, because of the possible risk of slow flow [26]. Of course, bradycardia (heart rate < 60 bpm) could be a problem during RA, beta-blockers can be continued, because the risk of slow flow was comparable between with and without beta-blockers [27].

Arrhythmia such as bradycardia or atrioventricular block sometimes happens in RA, especially during the treatment of RCA. Temporary pacing is a reliable option to continue procedures during arrhythmia. However, senior RA operators may not use temporary pacing by several reasons: [1] short ablation time may not induce sustained arrhythmia, [2] cough resuscitation may be effective for arrhythmia during RA [28], [3]there is a risk of ventricular perforation induced by temporary pacing catheter [29, 30]. Nevertheless, it would be a safe approach for junior RA operators to insert temporary pacing for specific lesions. Brady-arrhythmia can occur during RA to RCA, dominant left circumflex (LCX), left main trunk, and rarely left anterior descending artery (LAD) lesions. In this document, we recommend junior RA operators to consider temporary pacing for RA to RCA, dominant LCX, and left main trunk lesions.

## Guide catheter for RA

Although RA is possible either trans-radial, trans-femoral, or trans-brachial, RA operators should recognize the maximum burr size for each guide catheter size. A 6-Fr guide catheter can accommodate ≤ 1.75-mm burr, where as a 7-Fr guide catheter can accommodate ≤ 2.0-mm burr. When RA operators consider ≥ 2.15-mm burr, a 8-Fr guide catheter is necessary. However, if there is a severe tortuosity in a guide catheter, operators may feel a strong resistance during advancing the burr in the guide catheter, which results in the burr-size down. Moreover, if operators make side-holes in a guide catheter by themselves, such handmade side-holes may prevent the burr from advancing in the guide catheter. For junior RA operators, it is important to check coronary flow more frequently than senior RA operators to notice the occurrence of slow flow or perforation immediately, which would be easier in ≥ 7-Fr guide than in 6-Fr guide because of the large diameter of the drive shaft sheath (4.3 Fr).

The choice of guide catheter curves varies even among senior RA operators. Although the appropriate back-up support is important for stable procedures, the strongest back-up support, which is sometimes required for PCI to chronic total occlusion, would not be necessary for RA. The coaxial positioning of the guide catheter would be of utmost importance for successful RA. However, the strong back-up support can be a key to success in limited cases [31].

## Guidewire for RA

Two types of guidewire for RA have been commercially available: RotaWire floppy (Boston scientific, Marlborough, MA, USA) and RotaWire Extra-support (Boston scientific, Marlborough, MA, USA). Both RotaWires have 0.014-inch/0.36-mm spring tip and 0.009-inch/0.23-mm guidewire shaft. However, the length of the tapered segment is considerably different between RotaWire floppy and RotaWire Extra-support. RotaWire floppy has long tapered shaft (13 cm of < 0.0077-inch/0.20-mm shaft) and short spring tip [22 mm], whereas RotaWire Extra-support has short tapered shaft (5 cm of < 0.009-inch/0.23-mm shaft) and long spring tip [28 mm] [32]. For successful RA, it is important not to make a bend in RotaWires, because a bend in RotaWires substantially increase the friction force between burr and RotaWire. Therefore, the use of microcatheter is recommended to bring RotaWires to the target. RA operators should use the conventional 0.014-inch guidewire to cross the lesion, and then exchange the conventional guidewire to the RotaWire by using microcatheter. The guidance for selection of RotaWires is shown in Table 2.

**Table 2:** Guidance for selection of RotaWires

Characteristics or specific situations	RotaWire floppy or extra-support
Ability to ablate the severely calcified plaques (Ablation efficiency)	Extra-support > Floppy
Ability to straighten the tortuous coronary artery	Extra-support > Floppy
Ability to strengthen the back-up force in the system	Extra-Support > Floppy
When pre-intravascular imaging devices cross the lesion and provide sufficient information regarding the guidewire bias	Select either extra-support or floppy according to the information from imaging devices and angiography
When operators cannot judge the guidewire bias from angiography and/or intravascular imaging	Floppy first
When junior RA operators cannot understand which RotaWires are more suitable to the lesion	Floppy first
When the burr cannot cross the lesion, the exchange from floppy to extra-support	May work well, because of the change of the contact point. However, the strong guidewire bias may cause deep ablation
When the burr cannot cross the lesion, the exchange from extra-support to floppy	May work well, because of the change of the contact point

## Appropriate burr size

In early experiences with RA, big burrs were used to debulk the calcified plaques. However, a randomized trial comparing small burrs (burr/artery ratio of ≤ 0.7) with large burrs (burr/artery ratio of > 0.7) revealed that small burrs achieved similar immediate lumen enlargement and late target vessel revascularization compared with large burrs, but showed fewer complications [33]. European expert consensus document recommend burr/artery ratio of 0.6 [4], and North America expert consensus document recommend burr/artery ratio of 0.4–0.6 [3]. In this document, we recommend burr/artery ratio of 0.4–0.6 without intravascular imaging devices, and recommend to use intravascular imaging if RA operators aim to achieve burr/artery ratio of ≥ 0.6.

For lesion modification, single burr may be sufficient to facilitate stent delivery and stent expansion. However, second burr is sometimes necessary even for lesion modification. The guidance for second burr is summarized in Table 3.

**Table 3:** Guidance for use of the second burr

Size down or size up	Situations	Comments
Size down	When the first burr cannot cross the lesion, operators should size down with the second burr	This size down is important to prevent severe complications. It is better for junior RA operators to consider size down, when the burr could not cross the lesion after 4–6 RA sessions. If there are signs of slow flow such as chest pain or ECG changes, immediate size down should be considered
Size up	When operators aim to use the big burr (≥ 1.75-mm), start with the small burr (≤ 1.5-mm) for safety, and then size up to the big burr	Generally, the range of size up would be 0.25 mm to 0.75 mm. Junior RA operators should select the 0.25-mm or 0.50-mm size up
Size up	Operators started with the first burr, and checked intravascular imaging after the pass of the first burr. Intravascular imaging revealed the insufficient ablation, which recommended the size up	The thickness of calcification derived from OCT may be helpful to decide the necessity of size up. Before size up, operators should check signs of slow flow such as chest pain or ECG changes
Size up	Operators finished RA with the first burr, and then balloon (non-compliant balloon, scoring balloon, or cutting balloon) dilatation was tried. However, the lesion was not dilated sufficiently (typically dog-bone phenomenon), and then size up to the big burr	Careful manipulation of RotaWire is necessary, because there would be some dissections after balloon dilatation
Size up	During RA, no additional speed down was observed in spite of the forceful manipulation of the burr. Operators judged that the burr did not contact to the calcification adequately, and then sized-up to increase the contact area	No additional speed down in spite of forceful manipulation of the burr is the high-risk situation for burr entrapment or perforation

## Burr manipulation and rotation speed

A pecking motion (quick push-forward/pull-back movement of the burr) has been a standard burr manipulation in RA [4]. Although several burr manipulations have been conducted by RA experts, the common part of burr manipulation is to push-forward from the platform and pull-back the burr to the platform. The speed of manipulation varies widely among experts. Some experts prefer very quick, whereas other experts prefer very slow. Either speed is acceptable as long as the following points are considered: [1] operators should control the burr’s motion. If operators feel difficulty to control the burr’s motion, the speed may be too quick, [2] operators should avoid excessive rotational speed down, and [3] operators should not deactivate the system when the burr is in the middle of stenosis, which may result in the entrapment of the burr. Operators should deactivate the system when the burr was pulled-back to the platform.

Duration of individual runs is also important to prevent complications. Manufacturer recommend the duration of individual runs less than 30 s. In general, longer duration would be associated with greater amount of debris. For junior RA operators, short duration (e.g., ≤ l5–20 s) for a single session would be recommended. Furthermore, it is important to check the situations such as ECG and vital signs between the sessions.

Regarding the rotational speed, since manufacturer set the minimum speed as 140,000 rotations per minute (rpm), and the maximum speed as 190,000 rpm [34], this consensus document also recommends to use 140,000 to 190,000 rpm, and may consider to use > 190,000 rpm when operators feel difficulty to cross the lesion. There was a debate whether low rotational speed can reduce the risk of slow flow. Platelet aggregation was greater in high-speed (180,000 rpm) than in low-speed (140,000 rpm) in an early experiment in vitro [35], which has not been proved in vivo. Recently, a randomized control study comparing low-speed (140,000 rpm) with high-speed (190,000 rpm) revealed that the incidence of slow flow was similar between low-speed and high-speed [36]. Therefore, it is not reasonable to use low-speed for the prevention of slow flow. On the other hand, there were several interesting findings from Japan regarding the additional lumen gain in low-speed RA. Mizutani, et al. reported that the greater debulking area following low-speed (< 150,000 rpm) was confirmed by OCT [37]. Yamamoto, et al. also reported that the greater debulking area following very low speed (110,000 rpm) was confirmed by OCT [38]. However, Kobayashi, et al. reported that there were no additional lumen gain following low speed (120,000 rpm) [39]. Considering the above evidences, low speed (140,000 rpm) within the instructions for use could be an option to acquire additional lumen gain, but very low speed (< 140,000 rpm) should not be used, especially for junior RA operators.

The very-high speed (> 190,000 rpm) is sometimes used in Japan [8, 40]. A bench test showed that the RotaWire may be spinning under the maximum rotational speed [41], while the RotaWire theoretically would not spin during high-speed mode because of the internal brake and WireClip. The spinning of RotaWire may be associated with the guidewire failure [42, 43], which have not been proved in the large registry data.

## How to bring the burr to the platform

Before activating the burr, it is a key to success to bring the burr into the platform with keeping the RotaWire stable position. However, several troubles were frequently observed in the above process. The RotaWire could advance too deep or be pulled-back. The RotaWire may make a loop at the outside of the guide catheter, which can result in severe complications [44]. Although the manufacturer recommends to use the dynaglide mode only when operators remove the burr, not a few RA operators use the dynaglide mode when operators bring the burr to the platform. In this document, we would like to show the risk and benefit of both ways (using dynaglide or not) in Table 4. Because both ways have some disadvantage, operators need to understand both ways to avoid possible complications.

**Table 4:** Advantages and disadvantages of using dyna glide mode when operators bring the burr to the platform

	Using dynaglide mode	No dynaglide mode
Extent of resistance, when operators advance the burr	Less	Greater
RotaWire tends to advance more distally, when operators advance the burr	No	Yes
RotaWire tends to be pulled back, when operators advance the burr	Usually no, but possibly yes when the coaxiality of guide catheter was not maintained	Usually no, but possibly yes when assistant pulled the wire too much
Possibility of making a loop at the outside of the guide catheter	Very rare	Yes (very dangerous if operators could not notice it)
Combination between an operator and an assistant	Not important	Important (an assistant has to control the RotaWire during advancing the burr)
Jumping phenomenon, when operators activate the burr at the platform	Rare	Yes, therefore it is important to fix a nob at 1–2 cm apart from the end in advancer
Damage to the inner lumen of the guide catheter	Possible	No

## Rota cocktail

RA advancer has a saline infusion port. Although the instructions for use does not recommend to use any drugs into the saline bag, various drugs have been used to prevent slow flow. A representative combination of drugs was verapamil 10 mg [5 mg], nitroglycerin 5 mg (2.5 mg), heparin 10,000 unit (5000unit), and saline 1000 ml (500 ml). Another representative combination of drugs was nicorandil 24 mg [12 mg], nitroglycerin 5 mg (2.5 mg), heparin 10,000 unit (5000unit), and saline 1000 ml (500 ml). Two randomized studies compared nicorandil based cocktail with verapamil-based cocktail, and showed that the incidence of slow flow was significantly lower in the nicorandil based cocktail than in the verapamil-based cocktail [45, 46]. Preferred cocktail varied widely among RA experts, partly because intra-coronary injection of vasodilators such as nicorandil or nitroprusside was easily performed in the contemporary catheter laboratories. Either combinations of drugs are acceptable, as long as intra-coronary injection of vasodilators are available in a catheter laboratory. If only saline is used for RA, the activated coagulation time should be checked before RA to prevent possible thrombus formation.

## Imaging devices in RA

Imaging devices such as IVUS or OCT is useful in RA. In this document, we recommend to use IVUS or OCT before, during, and after RA. Since both IVUS and OCT have advantages and disadvantages, each device should be selected according to the purpose of RA in each case. The advantages and disadvantages of IVUS and OCT are summarized in Table 5. Although intravascular imaging before RA can provide useful information regarding appropriate burr size or RotaWire, intravascular imaging catheter may not cross the severely calcified lesions. If pre-RA imaging is critical for safe RA (e.g., ostium of left circumflex lesions), the use of guide-extension catheters may be considered to cross the lesion. For imaging device uncrossable lesions, small burrs (1.25-mm or 1.5-mm burrs) would be the choice. Either 1.25-mm or 1.5-mm burrs is to be decided at operator’s discretion. Sakakura, et al. compared the complications between 1.25-mm and 1.5-mm burrs for IVUS-uncrossable lesions, and showed the incidence of complications was comparable [47]. Moreover, the risk of complications was greater in imaging device uncrossable lesions than in imaging device crossable lesions [48]. Junior RA operators should be careful about those imaging device uncrossable lesions. Small balloon dilatation before RA can be an option for junior RA operators to prevent the severe complications.

**Table 5:** Comparison of intravascular imaging in RA between IVUS and OCT

	IVUS	OCT
Strong points	Since IVUS dos not need to eliminate red blood cells, operators can use IVUS safely when dissection or hematoma occurred following RA	OCT can provide the more detailed information regarding calcification such as thickness of calcification
Weak points	IVUS cannot prove the thickness of calcification	Difficult to use for ostial RCA or ostial LMT lesions Since OCT need to eliminate red blood cells, it is difficult to use OCT safely when dissection or hematoma occurred following RA Need to be careful for volume overload following multiple observations, especially for patients with low cardiac function May miss calcification when soft tissue buried the calcification
Judgement of guidewire bias	Accurate, but IVUS probe tends to separate from the guidewire when operators push an IVUS catheter. Before checking the guidewire bias, operators should pull the IVUS catheter a bit to correct the separation between the guidewire and IVUS probe	Accurate
Efficacy versus Safety	IVUS would increase the safety in RA. IVUS can be used in severe complications following RA, and the role of IVUS would be critical in some situations	OCT would increase the efficacy such as aggressive ablation in RA

## Endpoint of RA

Operators should set an appropriate endpoint of RA for each case. In general, when a burr crosses a lesion without any resistance and no additional speed down is observed, operators can finish RA unless operators consider burr size-up (conventional/classical endpoint of RA). In the contemporary PCI, the endpoint of RA may become more complex, owing to the development of IVUS/OCT. If operators use IVUS before RA, the crack in the napkin-ring calcification can be an endpoint of RA [7]. If operators use OCT before RA, the residual thickness of calcification or dissection can be an endpoint of RA [8, 49]. However, operators, especially junior RA operators, should not stick to the conventional/classical endpoint of RA, if there are signs of slow flow or other complications.

## Complications: slow flow

Slow flow is the most frequently observed complication following RA. The incidence of slow flow was approximately 5–20% [36, 46, 50], and varied widely among literatures, partly because the timing of judgement (just after RA or final shot) and the definition of slow flow were different among literatures. Lesion length and burr-to-artery ratio were reported as the determinants of slow flow [36]. For the prevention of slow flow, appropriate burr size, short ablation time, and gentle manipulation avoiding excessive speed down would be important to minimize the amount of debris caused by RA. Although there were no literatures, some experts prefer to flash saline for the prevention of slow flow during RA. Moreover, it is important to keep sufficient systolic blood pressure ≥ 120 mmHg (at least 100 mmHg). If patient’s cardiac function is normal, sufficient hydration is also important. If a patient shows low blood pressure under poor cardiac function, IABP may be considered. Noradrenaline diluted in saline is frequently used to keep blood pressure. If operators took a venous sheath from femoral vein as a rescue sheath, such sheath would be helpful to inject the drug immediately. Unlike slow flow during primary PCI, slow flow during RA is gradually developed (i.e., TIMI-3 flow to TIMI-2 flow, then TIMI-2 flow to TIMI-1 flow, etc.) as long as operators do not ablate lipid-rich plaques. Therefore, it is important to watch ST-elevations in ECG, which usually antecedent slow flow. If the TIMI-2 slow flow occurs, RA should be stopped temporarily until the TIMI-3 flow was restored. If blood pressure fall following slow flow, noradrenaline diluted in saline is injected to restore blood pressure. If noradrenaline did not work, the prompt insertion of IABP would be a next option. Intra-coronary vasodilators such as nitroprusside, nicorandil, and nitroglycerine are used to treat slow flow. Although there were no literatures comparing the efficacy among such vasodilators, nitroprusside may be the most potent vasodilator for slow flow [51]. Although nicorandil may be more effective than nitroglycerine [52], the rapid injection of nicorandil may provoke fatal arrhythmia or even cardiac arrest [53]. The use of microcatheters or double lumen catheters would be considered to minimize the risk of vasodilator-induced hypotension. Appropriate timing of intra-coronary vasodilators would be important to treat slow flow. Operators should check ECG or vital signs between sessions, and check the flow when the change of ECG was observed. In case of severe slow flow (TIMI-0), the use of thrombectomy catheter can be considered before the injection of intra-coronary vasodilators. The prevention and bailout for slow flow are summarized in Table 6.

**Table 6:** Prevention and bailout for slow flow

Prevention or bailout	Concept	Specific methods
Prevention	Do not make a large amount of debris in a session	Appropriate burr size Short ablation time Avoid excessive speed down
Prevention	Maintain sufficient blood pressure	Keep SBP ≥ 120 mmHg (at least 100 mmHg) Use of diluted noradrenaline Consider IABP when low SBP is derived from low cardiac function
Bailout	Immediate treatment is most important	Check the change of ST-segment, vital signs, and symptom (chest pain) between sessions Use of diluted noradrenaline if SBP fall Use of intra-coronary vasodilators such as nitroprusside

*SBP* systolic blood pressure, *IABP* intra-aortic balloon pumping

## Complications: perforation/rupture

Coronary perforation due to the burr is the most serious complication in RA, and the incidence of perforation in RA is approximately 1% [54, 55]. The risk of perforation highly depends on the lesion characteristics such as vessel tortuosity or eccentricity of calcification. Since the shape of each burr is ellipsoid [56], the RA burr cannot follow the sharply angulated vessel, which results in the greater risk of perforation. The risk of perforation is generally considered to be greater in an eccentric calcification such as calcified nodules than in a concentric calcification such as napkin-ring calcification. Thus, operators need to be careful for RA to an eccentric calcification. The selection of appropriate burr size and RotaWire should be important to prevent perforation, and the use of intravascular imaging devices would help operators to select appropriate burr size and RotaWire. If intravascular imaging shows the finding that the guide wire is pushing the normal vessel to distort the vessel structure, the risk of vessel perforation would be greater following RA. If intravascular imaging devices could not cross the lesion, small burrs (1.25-mm or 1.5-mm) should be the choice, especially for junior RA operators. In general, a RotaWire floppy would follow the vessel without distorting the vessel configuration, whereas a RotaWire Extra-support would follow the vessel with distorting the vessel configuration. Therefore, the route of the burr would be considerably different between RotaWire floppy and RotaWire Extra-support, which suggests the importance of the choice of RotaWires for the prevention of perforation. If the guidewire bias was difficult to anticipate by intravascular imaging or angiography, a RotaWire floppy would be the choice.

The bailout of perforation caused by RA is basically similar to that caused by PCI without RA except the fact that operators have to remove the RA system with keeping the RotaWire. If operators lost the RotaWire following massive perforation, there would be no guarantee of recrossing the guidewire. A Kusabi trapping balloon (Kaneka, Osaka, Japan) can be used for the retrieval of ≤ 1.5-mm burrs in ≥ 7-Fr systems [57]. A balloon catheter or a perfusion balloon catheter should be promptly delivered to the lesion to seal the blood flow toward pericardial space. Since the pericardiocentesis is probably necessary in perforation following RA, the preparation for pericardiocentesis is important in catheter laboratories using RA. Multiple covered stents may be necessary to seal the blood flow [58]. The use of guide extension catheter or the use of double guide catheters may be considered to facilitate the delivery of covered stents [59, 60], because there would be a risk of dislodgement of covered stents in severely calcified lesions. In the use of double guide catheters, a balloon via the first guide catheter can be used to seal bleeding during the preparation of covered stent via the second guide catheter, and the same balloon can be used as the distal anchor balloon to facilitate the covered stent delivery. Moreover, operators should contact to cardiovascular surgeons immediately just in case of unsuccessful percutaneous bailout. The prevention and bailout for coronary perforation are summarized in Table 7.

**Table 7:** Prevention and bailout for coronary perforation/rupture

Prevention or bailout	Concept	Specific methods/comments
Prevention	Risk assessment is of utmost importance for prevention of perforation following RA	Greater risk in lesions with an angulation Risk of perforation is greater in eccentric calcification than in concentric calcification
Prevention	Use appropriate burr size, and select appropriate RotaWires	Do not push the burr too much, just deliver the bur to the lesion Small burrs (≤ 1.5-mm) would be the choice for the high- risk lesions Interpretation of guidewire bias derived from intravascular imaging would be important to select appropriate RotaWires
Bailout	Keep the RotaWire within the lesion, when perforation occur	Do not be panic. Remove the Rota system with keeping the RotaWire within the lesion Covered stents and pericardiocentesis would be necessary in most cases Contact cardiovascular surgeons immediately in case of unsuccessful percutaneous bailout

## Complications: burr entrapment

**Fig. 1:**
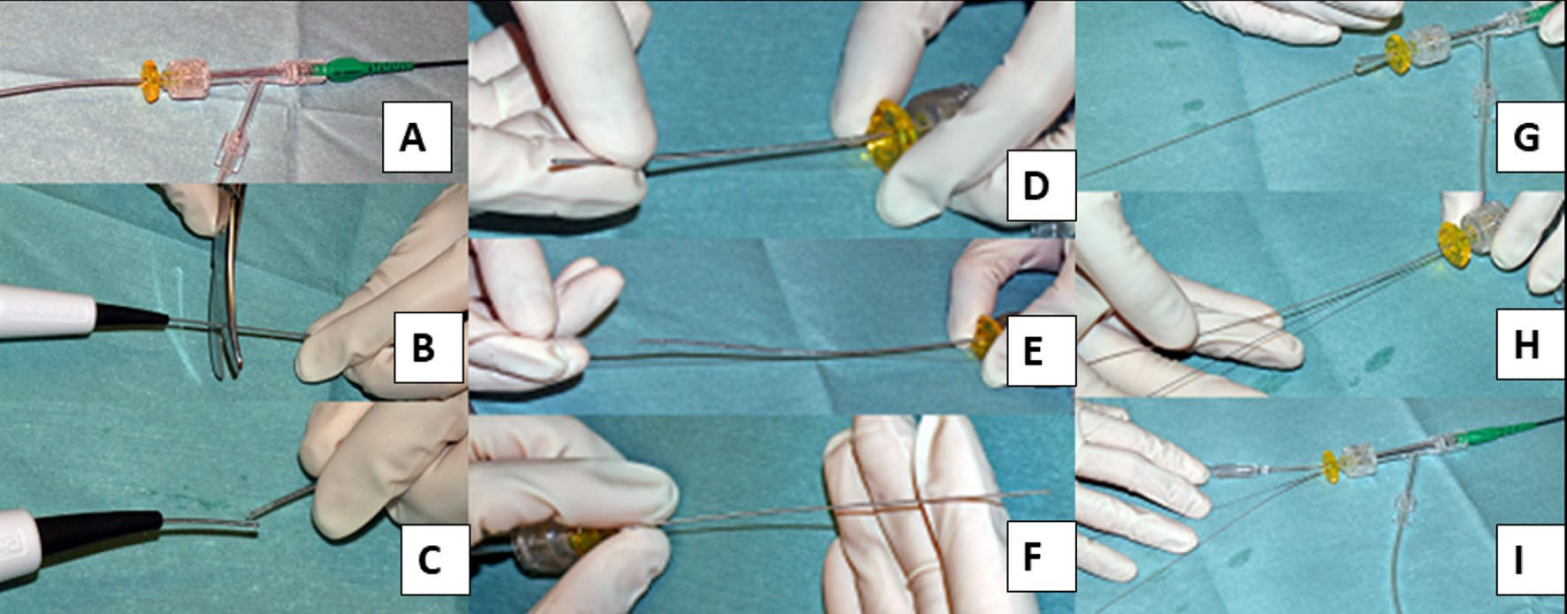
How to cut and pull the drive shaft sheath. Panel **a**: a RA burr (1.25-mm) was inserted into a 6-Fr guide catheter via a Y connector. Panel **b**, **c**: the drive shaft, drive shaft sheath, and RA wire were cut together near the advancer. Panel b, **e**: the drive shaft sheath was pulled back and removed. Panel **f**: after the drive shaft sheath was removed, the drive shaft remained in the same position. Panel **g**, **h**: a guide wire (0.014 in) passed through the guide catheter via an inserter and Y-connector. Panel **i**: a 2.5 × 15 mm conventional balloon easily passed through the guide catheter. This figure was reproduced with the permission from Sakakura, et al. [63]

Burr entrapment is a unique complication in RA, and the incidence of burr entrapment is not derived from multicenter registries, but is available from single center studies ranging 0.4%–0.8% [9, 61]. Burr entrapment can occur from several mechanisms. One is called as “Kokesi phenomenon” that the burr was trapped in the distal portion of the proximal narrowing [62]. The mechanism of Kokesi phenomenon is considered to be that a friction heat enlarges the orifice and the coefficiency of friction in motion is smaller than that of friction at rest [62]. This type of burr entrapment may occur following forceful manipulation with small burrs. Another mechanism is the burr entrapment related to the vessel angulation. Since the shape of the burr is ellipsoid and the diamond coating is not available at the tail of the burr, the burr can be trapped by non-massive calcification at the site of angulation. To prevent burr entrapment, operators need to be careful about rotational speed reduction, sound of ablation, and resistance during the burr manipulation. If operators encounter the burr entrapment, it is important to assess the situation calmly. The presence of antegrade flow, ST-segment elevations in ECG, and patient’s chest pain should be evaluated. If there is no antegrade flow beyond the entrapped burr, the percutaneous bailout would be very difficult, and be limited to experienced senior RA operators. In the meantime, operators should contact cardiovascular surgeons to discuss the surgical bailout. If the antegrade flow is present without ST-segment elevations, operators would have a time to consider the percutaneous bailout techniques. Although there have been several percutaneous bailout techniques in literatures [63–69], the main difference among various techniques was whether to use additional guide catheter (double guide catheters) or not (single guide catheter). If operators selected the double guide systems, operators would insert the conventional guidewire from the second guide catheter, and then would try to dilate the proximal part of the entrapped burr using a balloon [64]. If operators selected the single guide system, the next step would depend on the guide catheter size (≥ 8 Fr or ≤ 7 Fr). If operators used a ≥ 8Fr guide system, operators would insert the conventional guidewire, and then would try to dilate the proximal part using a balloon. However, if operators used a ≤ 7 Fr guide system, operators need to cut and pull the drive shaft sheath (Fig. 1) [63], because a ≤ 7 Fr guide catheter cannot accommodate the drive shaft sheath, guidewire, and balloon catheter together. After pulled out the drive shaft sheath, operators can try to dilate the proximal part using a balloon. Once operators pulled out the drive shaft sheath, operators can use inner catheters such as guide extension catheters [65, 68, 69]. Of course, operators should recognize the possibility of unsuccessful percutaneous bailout, and need to prepare the massive perforation or severe dissection following the burr retrieval [70]. The prevention and bailout for burr entrapment are summarized in Table 8.

**Table 8:** Prevention and bailout for burr entrapment

Prevention or bailout	Concept	Specific methods/comments
Prevention	Risk assessment is of utmost importance for prevention of perforation following RA	Do not push the burr too much, just deliver the bur to the lesion Greater risk in lesions with an angulation Be careful about rotational speed deceleration, sound of ablation, and resistance during the burr manipulation
Prevention	Do not inactivate the burr in the middle of the calcified stenosis	There is no diamond coating at the tail of the burr Moderate stenosis at the proximal of the target can be a cause of burr entrapment
Bailout	It is important to assess the situation such as the presence of antegrade flow, calmly	Do not activate the burr after burr entrapment Bailout techniques are divided to single guide bailout or double guide bailout Contact cardiovascular surgeons immediately in case of unsuccessful percutaneous bailout

## Complications: transection of the RotaWire

The transection of the RotaWire is a rare complication in RA, and the incidence of transection of the RotaWire is not derived from multicenter registries, but may be approximately 0.4%–1% [43, 61]. There are two types of the transection of the RotaWire: one is the transection at the radiopaque part of the RotaWire, and the other is the transection at the radiolucent part of the RotaWire. The transection of the radiopaque part is easy to notice. If there were the transection of the radiopaque part of the RotaWire, operators should exchange the broken RotaWire to the new one. Since the retrieval of a transected fragment of the RotaWire would be similar to that of the conventional guidewire, operators might try retrieval procedures such as twin guidewire method [71]. If the transected fragment of the RotaWire located at the far distal segment of the treated vessel, operators might leave it at the distal segment rather than retrieval. On the other hand, the transection of the radiolucent part of the RotaWire is very difficult to notice. If operators could not notice the transection of the radiolucent part of the RotaWire, operators would have a vessel perforation [72–74]. Moreover, there would be a greater risk in retrieval of a transected fragment of the RotaWire, if the transection occurred at the radiolucent part. Because the proximal part of the transected fragment of the RotaWire would be sharp, the invisible sharp fragment of the RotaWire may damage to the proximal vessel wall, even to the aortic wall during the retrieval. IVUS may be helpful to identify the invisible fragment.

Since the incidence of transection of the RotaWire is low, the causes of transection have not fully understood. Because the metallic fatigue would be the possible cause of transection, it may be important to avoid the continuous contact between the burr and the specific part of the RotaWire. If the RotaWire kinked at the outside of the guide catheter, the transection of RotaWire would happen when the burr advanced over the kinked RotaWire [44]. Furthermore, the WireClip Torquer (Boston Scientific, Marlborough, MA, USA) should be equipped with the RotaWire during the dyna-glide mode as well as the high-speed mode to avoid the spinning of the RotaWire, which could be a cause of transection of the RotaWire.

## Complications: disconnection of the burr

Disconnection of the burr is very rare complications, and there have been no literatures mentioning the incidence of disconnection of the burr. The exact reasons or mechanisms of disconnection of the burr have not been specified. Even senior RA operators with abundant experiences may not or may have a few cases with this complication. Theoretically, the disconnection of the burr could happen when operators activate the burr after the burr was entrapped, or when operators advanced the burr in spite of strong resistance within the stented segment or angulated segment. Operators may notice the disconnection of the burr by the loss of coordination between the burr motion and the nob motion. The percutaneous bailout may be possible if the RotaWire is not transected, because the 0.014-inch spring tip of the RotaWire may work as the anchor. Imamura S, et al. reported a case of the disconnection of the burr, which was successfully treated percutaneously [75]. In their case report, since the simple pull-back using a guide extension catheter did not work, they sandwiched the disconnected burr between the inflated balloon and the guide extension catheter, and then pulled back together [75]. However, surgical bailout should be considered to this rare complication [76].

## Specific lesions: RCA ostial lesions

Since clinical outcomes of RCA ostial lesions have been unsatisfactory for decades [77], RA has been considered to be a good indication for RCA ostial lesions with severe calcification [23]. However, a good indication does not necessarily mean an appropriate lesion for junior RA operators. Sakakura, et al. reported that the excessive speed reduction during RA was significantly associated with RCA ostial lesions [24], which suggests the difficulty of RA for RCA ostial lesions. There are several reasons why RA for RCA ostial lesions are difficult. First, it is impossible to insert the guide catheter to RCA coaxially. Operators would try to keep guide catheter coaxial to RCA in aorta. Second, coaxiality cannot be confirmed by LAO view, which is a standard view in RA for RCA ostial lesions. Coaxiality is usually confirmed by RAO view in conventional PCI. If the guide catheter can engage to the RCA, coaxiality would be kept once operator check in the RAO view. However, if the guide catheter cannot engage to the RCA, coaxiality would not be kept during procedures. Because it is difficult to check the burr motion in RAO view due to the angiographical shortening of the RCA ostial lesion, the frequent switch between RAO view and LAO view may be necessary to keep coaxiality during RA. Another option is to use straight cranial view, which allows operators to check coaxiality without angiographical shortening of the RCA ostial lesion.

**Fig. 2:**
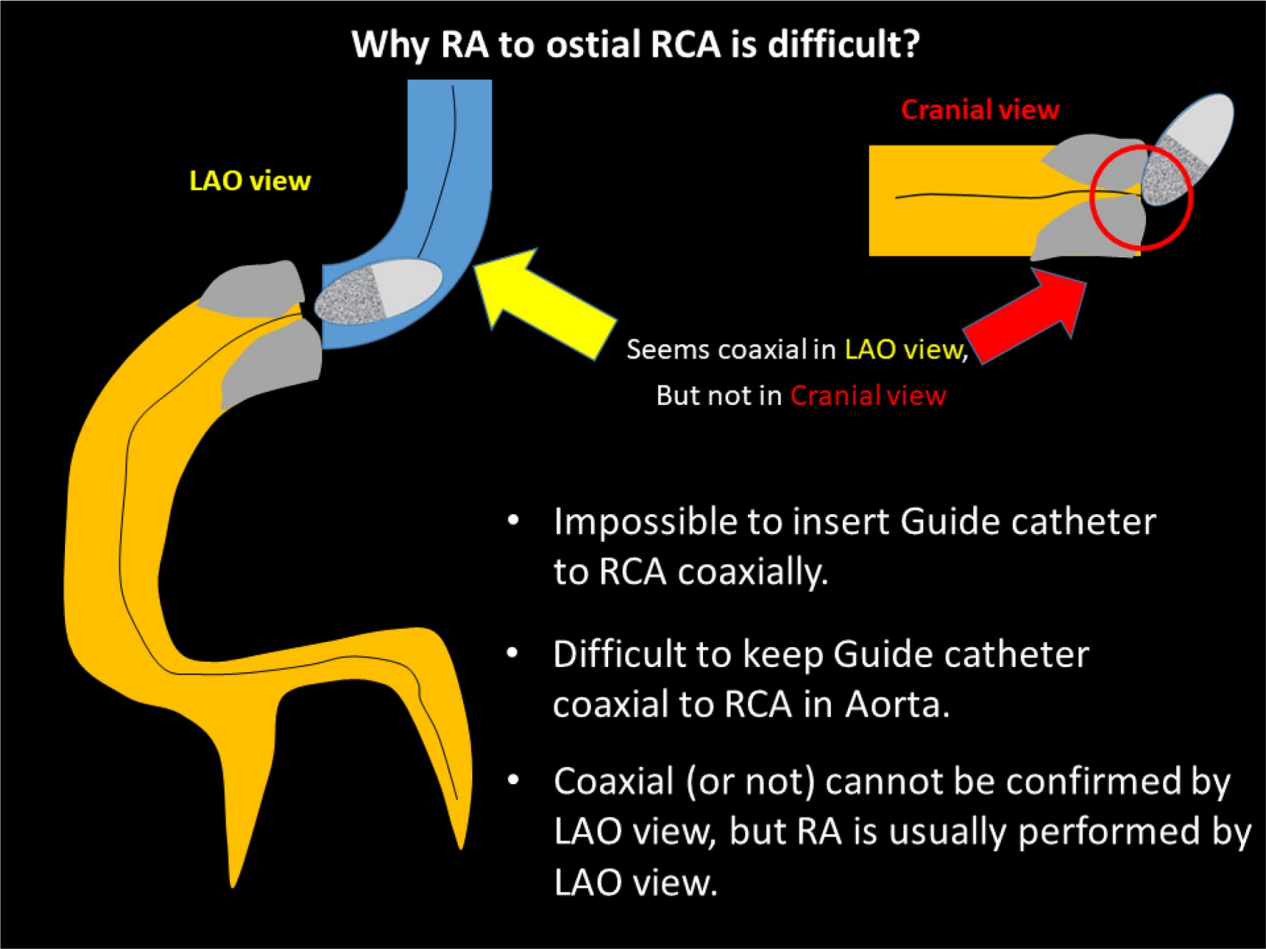
Why RA to ostial RCA is difficult?

The selection of the RotaWire for RCA ostial lesions is also important. The RotaWire Extra-Support may be preferable when operators intentionally remove the guide catheter from the RCA ostium, whereas the RotaWire Extra-Support may be dangerous when operators cannot take a coaxial position. IVUS may help to estimate the risk of RA, especially when operators could not take a coaxial position. Although an IVUS catheter may not cross the lesion before RA, an IVUS catheter would cross the lesion after the crossing of small burrs. IVUS should be tried before using the big burrs for RCA ostial lesions. Furthermore, there may be an additional risk of cerebral infarction following RA for RCA ostial lesions. It may be important for the prevention of cerebral infarction to use small burrs for an initial attempt to minimize the size of debris. Figure 2. summarized the why RA to ostial RCA is difficult. RA for RCA ostial lesions is not recommended for junior RA operators without senior RA operator’s back-up.

## Specific lesions: LCX ostial lesions with substantial bending

**Fig. 3:**
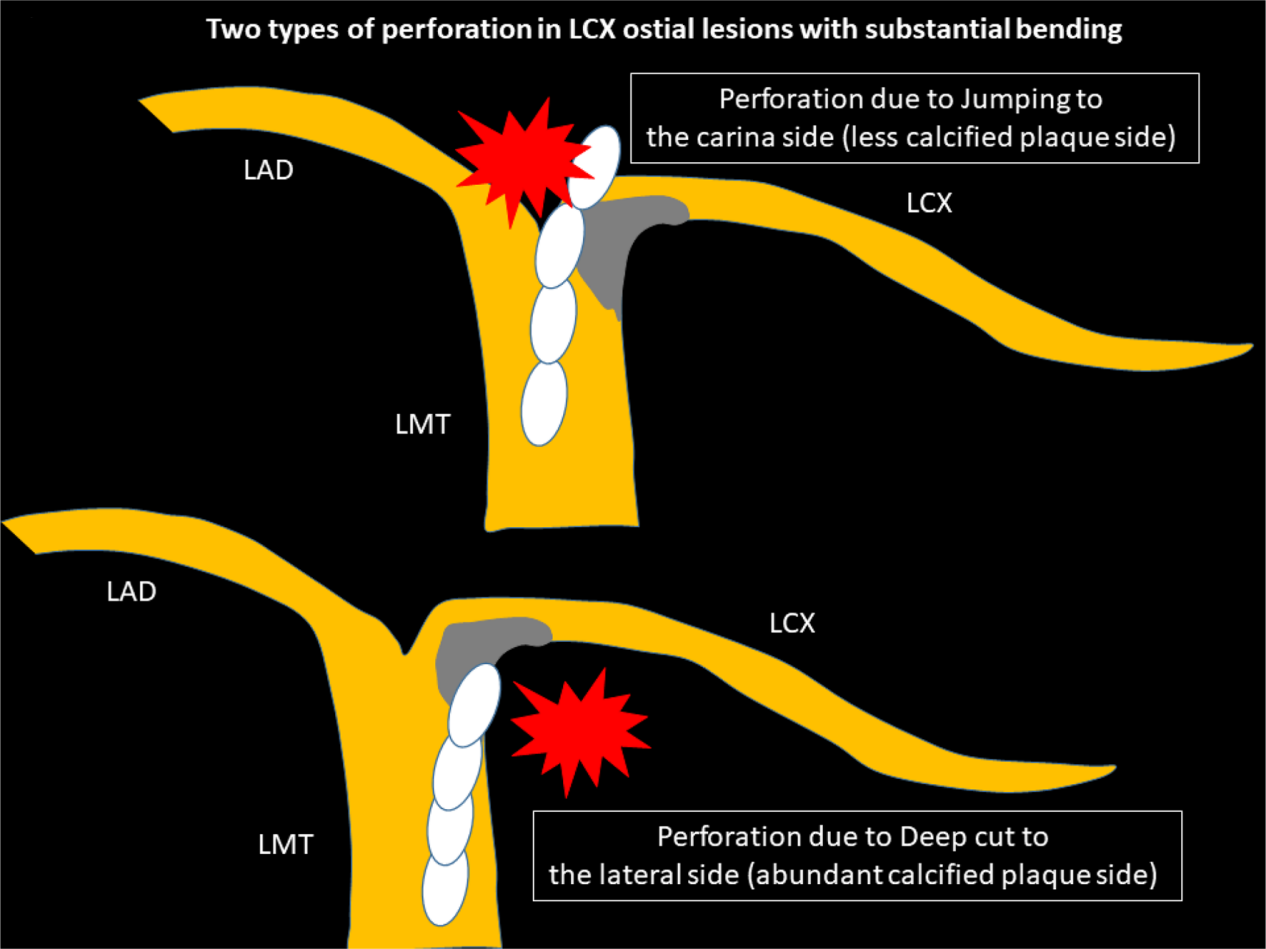
Schema of two types of perforation in LCX ostial lesions with substantial bending

Severely calcified left circumflex (LCX) ostial lesions with substantial bending may be the highest risk lesion in RA. The key to success would be the interpretation of pre-procedural intravascular imaging. If the pre-procedural imaging device could cross the lesion and provide sufficient information including the guidewire bias, operators could select appropriate RotaWires and burrs. However, if the pre-procedural imaging device could not cross, operators need to select RotaWires and burrs from the only angiographical information. In general, atherosclerotic plaques are observed in the lateral wall, whereas atherosclerotic plaques are spared in the flow divider regions (carina) [78]. If severe eccentric calcification was observed in the lateral wall of the LCX ostium, there would be a risk of perforation in the carina side of the LCX ostium due to the jumping of the burr. Operators would try to ablate the lateral wall of the LCX ostium to avoid perforation in the carina side. However, if operators ablated the lateral wall too much, there would another risk of perforation in the lateral wall side due to the deep cut. Two types of perforation in LCX ostial lesions with substantial bending are illustrated in Fig. 3. Operators need to select appropriate burr size, RotaWires, and burr motion to prevent the above two types of perforation. In angiography, it would be important to evaluate the actual contact point between the calcification and the RotaWire using multiple projections. Although low-dose radiation angiography is important for patient’s safety, excessive low-dose radiation angiography may sacrifice the visibility of RotaWires or calcification. Since the visibility of RotaWires or calcification is the cornerstone for high-risk RA, operators should set the radiation dose not to prevent the visibility of RotaWires or calcification. Moreover, if there would be a perforation in the LCX ostium, the percutaneous bailout would be difficult, because the implantation of covered stents may occlude the left anterior descending artery (LAD). Therefore, the indication as well as the strategy of RA to the LCX ostium should be carefully discussed. RA for LCX ostial lesions with substantial bending is not recommended for junior RA operators without senior RA operator’s back-up.

## Specific lesions: unprotected left main lesions

RA to the unprotected left main lesions requires special attention, because slow flow may cause hemodynamic collapse. However, Fuku et al. compared clinical outcomes of left main PCI between with RA (*n* = 108) and without RA (*n* = 1091), and showed the similar rate of complications in left main PCI between with RA and without RA [79], suggesting the acceptable safety in PCI to left main lesions using RA. In clinical practice, it would be important to start with small burrs (≤ 1.5-mm burr) to prevent fatal slow flow. However, the ≤ 1.5-mm burr may be too small as the final burr size considering the vessel diameter of left main lesions. It would be important to use IVUS/OCT to select an initial burr size and final burr size. If the severe calcified plaques exist at the ostium of left main, the procedure would be more complex as compared to calcified plaques at the middle of left main. In that situation, the stabilization of guide catheter at co-axial position within aorta would be the key to success like the ostium of RCA lesions. Furthermore, the use of IABP and/or temporary pacing should be considered. RA for left main lesions is not recommended for junior RA operators without senior RA operator’s back-up.

## Specific lesions: stent ablation

Stent ablation is applied to the restenotic lesions caused by under-expanded stents. Okamura, et al. described a case of stent ablation to treat restenosis lesions due to crushing of a sirolimus-eluting stent [80]. In their report, they performed an experimental study and found that the size of the metallic particles generated during RA of stent struts was 5.6 ± 3.6 μm, which suggested the safe size in a human body [80]. Tips of stent ablation are [1] to select the appropriate burr size, [2] to ablate only stent struts first, [3] and then ablate the calcification behind stent struts using the second burr (size up). Since the risk of burr entrapment is greater during stent ablation, gentle manipulation with gradual size-up would be important. Although stent ablation might be a single solution for the restenostic lesions caused by under-expanded stents, the long-term outcomes was not satisfactory [81–83]. Coronary lithoplasty may be another option for stent underexpansion due to severe calcification [84], while sufficient follow-up data are not available yet. Stent ablation is not recommended for junior RA operators. Furthermore, on-site surgical back-up may be need because of greater risk of burr entrapment, when operators perform stent ablation using big burrs.

## Specific lesions: diffuse long lesions

**Fig. 4:**
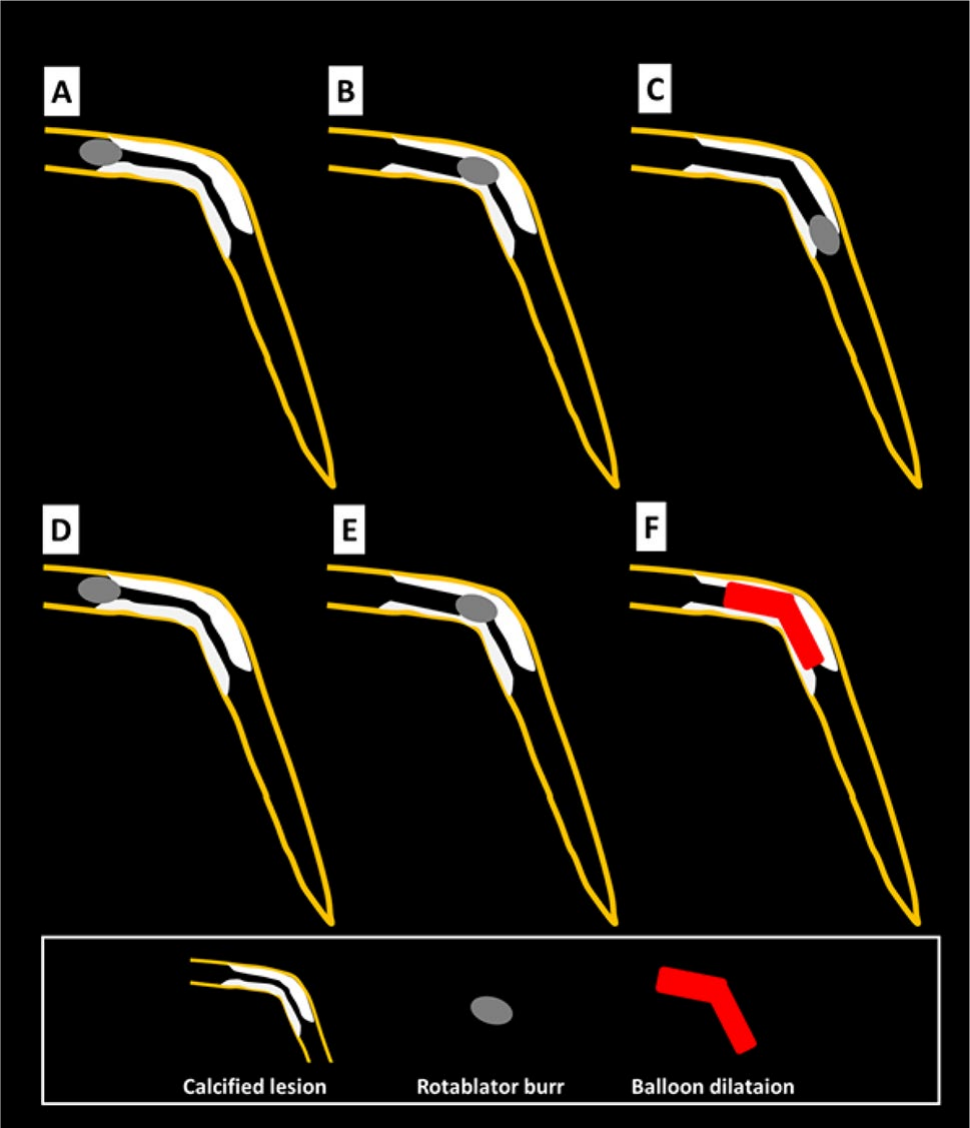
Schema of conventional and halfway rotational atherectomy. Panels (**a**), (**b**), and (**c**) illustrate the conventional rotational atherectomy, whereas panels (**d**), (**e**), and (**f)** illustrate the halfway rotational atherectomy. **a** The burr positioned just before the calcified lesion. **b** The burr ablated the proximal segment of the calcified lesion. **c** The burr ablated the full segment of the calcified lesion. **d** The burr positioned just before the calcified lesion. **e** The burr ablated the proximal segment of the calcified lesion. **f** Balloon dilatation was performed for the rest of the calcified lesion. This figure was reproduced with the permission from Sakakura, et al. [87]

RA was frequently performed for diffuse long lesions, and the long-term outcomes of long lesions treated by RA were comparable to those of short lesions treated by RA [85]. However, RA for diffuse long lesions may be difficult for junior RA operators, especially when there is an angle in the middle of diffuse long lesions. Sakakura, et al. reported the utility of halfway RA especially for lesions with an angle [86, 87]. In brief, the operator does not advance the burr beyond the angle within the lesion to avoid burr entrapment or vessel perforation, and balloon dilatation is performed beyond the angle after RA (Fig. 4). Halfway RA may be useful especially for junior RA operators. If halfway RA did not work (e.g., balloon could not dilate the distal calcified lesion), switch from halfway RA to conventional RA should be considered. Although the other option could be to use the guide extension catheter, RA beyond the guide extension catheter is not straightforward. Another important aspect in RA for diffuse long lesions is to check the speed down of rotational speed. Although the excessive speed down during RA should be avoided, absence of reasonable speed down may mean that the burr does not contact to calcification adequately. If an operator pushes the burr too much in the absence of speed down, there would be substantial risk of burr entrapment or vessel perforation. Therefore, an operator may change the RotaWires to facilitate the contact between the burr and the calcified plaques, switch to balloon dilatation (resultantly halfway RA), or rarely burr size-up to increase the contact area in the absence of reasonable speed down [56, 88].

**Fig. 5:**
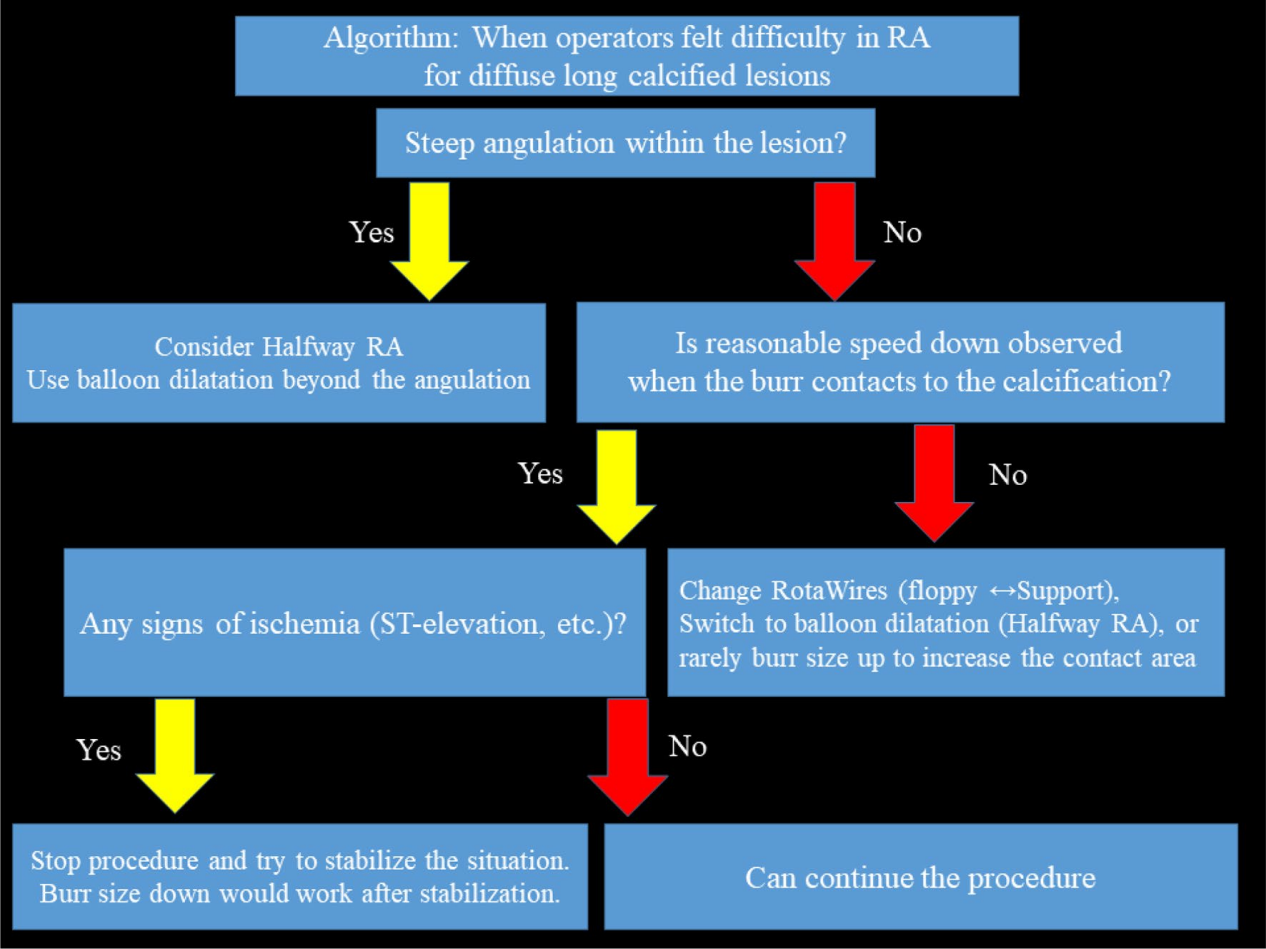
Algorithm: when operators felt difficulty in RA for diffuse long calcified lesions

Operators should also take care of ischemia during RA to the diffuse long lesions, because the risk of slow flow is greater in the diffuse long lesions than the short lesions [36]. If operators noticed the signs of ischemia in the middle of diffuse long lesions, size down of the burr would be the reasonable choice. Another option would be to take a short break with removing the burr from the coronary artery to stabilize coronary flow and vital signs. If coronary flow is restored and vital signs are stabilized, operators can safely resume procedures. This document suggests an algorithm for disuse long lesions, especially for junior RA operators (Fig. 5).

The maximum working range of the burr is approximately 70 mm. Operators usually set the nob at 1–2 cm apart from the end, and set the platform at 1–2 cm proximal from the lesion. Thus, the actual working range of the burr is approximately 30–50 mm. If the length of the target lesion was beyond 30–50 mm, operators need to move the platform during the procedure. Although there were few literatures regarding how to move the platform during the procedure, there were several ways among RA experts. The most important point is to ablate the proximal part of the diffuse long lesion sufficiently before moving the platform. Only one pass would not be enough. Several passes would be necessary until no additional speed down was observed. Some experts prefer to size up the burr and ablate the proximal part of the diffuse long lesion using big burrs to make a stable platform. After operators ablated the proximal part of the diffuse long lesion sufficiently, operators could move the platform more distally. Some experts prefer to use dynaglide mode to move the platform. Sliding sheath technique, in which operators park the burr to the distal end and then bring the advancer to follow the burr, may be used by some experts. Since the outer diameter of the drive shaft sheath is 4.3 Fr (1.43 mm), the ablation by the burr-1.25 mm may not be enough to move the platform.

## Conclusions

In this document, we provided the Japanese style RA, which uses intravascular imaging devices to achieve the maximum efficacy without sacrificing the safety. Our recommendation focused on mainly junior RA operators, but would be helpful for senior RA operators for their more advanced procedures.
