# Correction to: Clinical expert consensus document on rotational atherectomy from the Japanese association of cardiovascular intervention and therapeutics

**DOI:** 10.1007/s12928-020-00749-0

**Published:** 2021-01-03

**Authors:** Kenichi Sakakura, Yoshiaki Ito, Yoshisato Shibata, Atsunori Okamura, Yoshifumi Kashima, Shigeru Nakamura, Yuji Hamazaki, Junya Ako, Hiroyoshi Yokoi, Yoshio Kobayashi, Yuji Ikari

**Affiliations:** 1 Division of Cardiovascular Medicine, Saitama Medical Center, Jichi Medical University, 1-847 Amanuma, Omiya, Saitama City, 330-8503 Japan; 2 Department of Cardiology, Saiseikai Yokohama City Eastern Hospital, Yokohama, Japan; 3 Department of Cardiology, Miyazaki Medical Association Hospital, Miyazaki, Japan; 4 Division of Cardiology, Sakurabashi Watanabe Hospital, Osaka, Japan; 5 Division of Interventional Cardiology, Cardiovascular Medicine, Sapporo Cardio Vascular Clinic, Sapporo Heart Center, Sapporo, Japan; 6 Cardiovascular center, Kyoto Katsura Hospital, Kyoto, Japan; 7 Division of Cardiology, Ootakanomori Hospital, Kashiwa, Japan; 8 Department of Cardiovascular Medicine, Kitasato University School of Medicine, Sagamihara, Japan; 9 Department of Cardiology, Fukuoka Sanno Hospital, Fukuoka, Japan; 10 Department of Cardiovascular Medicine, Chiba University Graduate School of Medicine, Chiba, Japan; 11 Department of Cardiology, Tokai University School of Medicine, Isehara, Japan

## Correction to: Cardiovascular Intervention and Therapeutics 10.1007/s12928-020-00715-w

The article Clinical expert consensus document on rotational atherectomy from the Japanese association of cardiovascular intervention and therapeutics, written by Kenichi Sakakura, Yoshiaki Ito, Yoshisato Shibata, Atsunori Okamura, Yoshifumi Kashima, Shigeru Nakamura, Yuji Hamazaki, Junya Ako, Hiroyoshi Yokoi, Yoshio Kobayashi, Yuji Ikari, was originally published electronically on the publisher’s internet portal on 20 October 2020 without open access. With the author(s)’ decision to opt for Open Choice the copyright of the article changed on 17 December 2020 to © The Author(s) 2020 and the article is forthwith distributed under a Creative Commons Attribution 4.0 International License, which permits use, sharing, adaptation, distribution and reproduction in any medium or format, as long as you give appropriate credit to the original author(s) and the source, provide a link to the Creative Commons licence, and indicate if changes were made. The images or other third party material in this article are included in the article’s Creative Commons licence unless indicated otherwise in a credit line to the material. If the material is not included in the article’s Creative Commons licence and your intended use is not permitted by statutory regulation or exceeds the permitted use, you will need to obtain permission directly from the copyright holder. To view a copy of this licence, visit http://creativecommons.org/licenses/by/4.0/.

The original article has been corrected.

